# A rare case report of acyclovir-induced immune thrombocytopenia with tongue hematomas as the first sign, and a literature review

**DOI:** 10.1186/s40360-017-0120-2

**Published:** 2017-03-07

**Authors:** Xiaowei Hong, Xiaoqian Wang, Zhiyong Wang

**Affiliations:** 1 Nanjing Stomatological Hospital, Medical School of Nanjing University, Nanjing, 210008 People’s Republic of China; 2 State Key Laboratory of Oral Diseases, National Clinical Research Center for Oral Diseases, West China Hospital of Stomatology, Sichuan University, Chengdu, 610041 People’s Republic of China

**Keywords:** Acyclovir, Thrombocytopenia, Varicella-zoster virus, Tongue hematoma, Case report

## Abstract

**Background:**

Acyclovir has been widely used to treat infections caused by herpes simplex virus (HSV) and varicella zoster virus (VZV). The common adverse effects of this drug include nausea, diarrhea, headache, dizziness and mental changes. The immune thrombocytopenia induced by acyclovir is rare.

**Case presentation:**

A 67-year-old Chinese male who was given acyclovir 5 mg kg^−1^ 8 hourly intravenously for treatment of VZV infection developed severe thrombocytopenia with fist sign in oral cavity within 10 days of starting using acyclovir. The patient’s condition was improved by stopping using acyclovir and further supportive treatment. The acyclovir-dependent platelet antibody test showed positive results, which implicated acyclovir as the causative agent. The final definitive diagnosis of acyclovir-induced immune thrombocytopenia was established basing on the time correlation between the start of using acyclovir and the onset of symptoms of thrombocytopenia, combining with excluding of other common causes of thrombocytopenia.

**Conclusion:**

There have been few reports of acyclovir-induced immune thrombocytopenia. This is the first case report and literature review of acyclovir-induced immune thrombocytopenia, with tongue hematoma as the first sign. Dentists should never overlook this rare adverse effect of acyclovir, as a rapid and appropriate treatment may prevent further severe life-threatening complications.

## Background

Acyclovir, an acyclic purine nucleoside analogue, has been widely used because of its highly potent prohibitive properties for infections caused by HSV and VZV [1, 2]. Acyclovir has minimal toxicity to normal host cells, because the drug is only adsorbed by the virus infected cells [3]. Severe adverse effect like neurotoxicity, kidney disorders and psychiatric was not common, mostly related to high dose intravenous administrations [4–6]. Only a few reports of acyclovir-induced myelosuppressive have been published [7–9], and so far only two case reports of acyclovir-induced thrombocytopenic purpura have been reported [3, 10]. Our patient, a 67-year-old Chinese man who received acyclovir for the VZV infection, presented with oral hematomas as the first sign. We believe this is the first well-documented case report of acyclovir-induced immune thrombocytopenia as the first sign in oral cavity.

## Case presentation

A 67-year-old man who was retired presented to the Oral Maxillofacial Surgery Department of Nanjing Stomatology Hospital, Nanjing, Jiangsu, China, with a 10-day history of a “lump” on the left part of his tongue. The patient felt pain when eating, and the “lump” gradually grew in size. There was a similar “lump” on the right side of his tongue, which then regressed with no treatment. Before having the “lump” on his tongue, the patient had sought treatment at a general hospital, and was given intravenous acyclovir 5 mg kg^−1^ 8 hourly for 7 days because of a VZV infection. The patient denied fever, hematochezia, melena, hemoptysis, hematuria, and neurologic symptoms. He had no history of bibulosity, smoking, systemic diseases, or drug allergy. His family history, social history, and oral treatment history were unremarkable. No other drugs were taken during the treatment of the varicella-zoster virus infection.

### Clinical examination

**Fig. 1:**
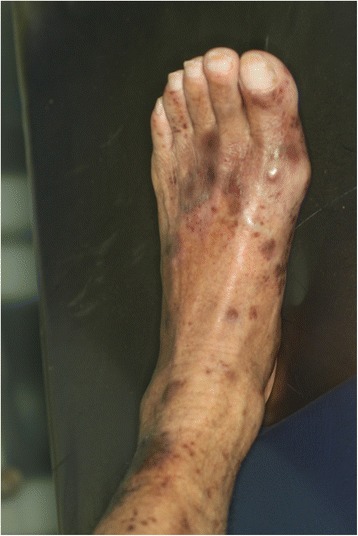
Extra-oral examination of patient: purple spots on patient’s legs and feet

Extraoral examination: There was no enlargement or change in texture of the maxillofacial and neck lymph nodes, and no limitation of mouth opening (mouth opened to 40 mm). No swelling or deformity of the maxillofacial area and neck were noted. There were several ecchymoses on the patient’s legs and feet (Fig. 1).

**Fig. 2:**
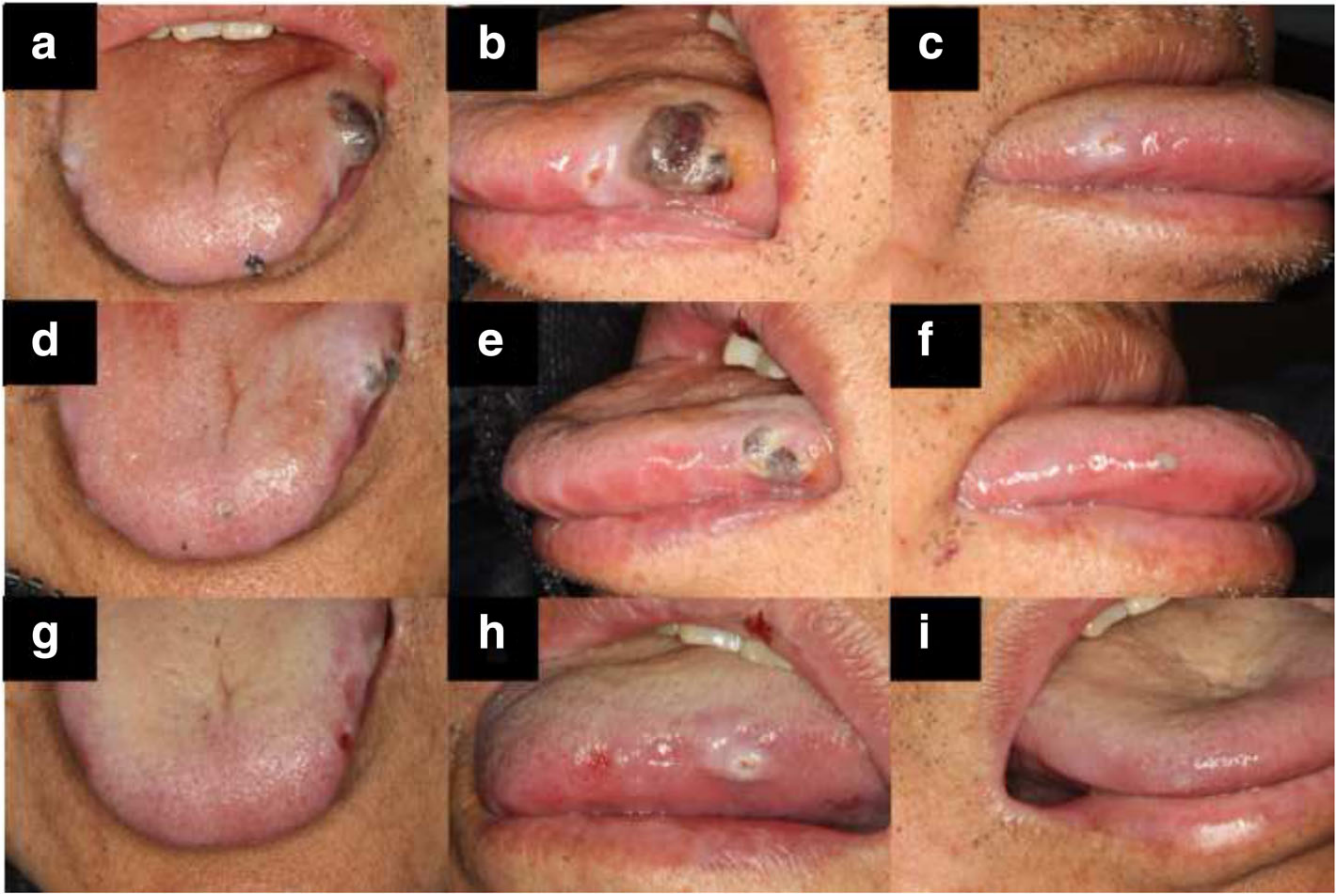
Intraoral examination of the first visit (**a**-**c**), the second visit (**d**-**f**), and the third visit (**g**-**i**)

Intraoral examination: There were three main lesions in the patient’s mouth. The first lesion appeared on the left margin of the tongue, a dark purple hematoma, 1.5 cm × 1 cm in size, with a medium texture and hard base. The lesion was painful on compression, but did not change color when pressed. Bleeding at the margin of the lesion was detected (Fig. 2a and b). The second lesion was on the left side of the tongue tip, showing a dark purple spot 0.3 cm in diameter (Fig. 2a). It was not painful on compression. The third lesion was a 0.5 cm × 0.5 cm white plaque with a bleeding spot at the center (Fig. 2c), located on the right margin of the tongue, and eliciting no pain on compression.

### Laboratory test

**Fig. 3:**
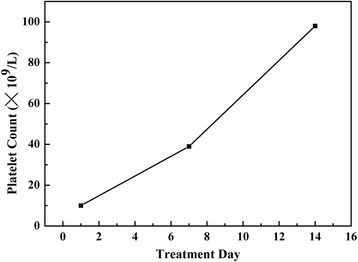
Time course of the patient’s platelet count after starting the treatment

A complete blood count, performed on the day of presentation, showed a hemoglobin concentration of 120 g/L (120–160 g/L), a white blood cell count of 8.75 × 10^3^/mm^3^ (4.6–10.2 × 10^3^/mm^3^) with 58.1% neutrophils (50–70%), 25.8% lymphocytes (20–40%), and 9.6% monocytes (2–10%); the platelet count was 10 × 10^9^/L (100–300 × 10^9^/L) (Fig. 3). Other laboratory tests showed normal liver function, a normal electrolyte profile, and normal haptoglobin, bilirubin (total and direct), and lactate dehydrogenase (LDH) levels. Creatinine clearance was 59.2 mL/min. Coagulation studies were normal. Tests for cytomegalovirus, hepatitis A and B viruses, and Epstein–Barr virus yielded negative results. The result for platelet factor 4 (PF4)/heparin antibodies using enzyme-linked immunoassay (ELISA) was positive. The following serotonin-release assay (SRA) yielded a negative result. ELISA was also applied for detection of acyclovir-dependent platelet antibodies in vitro, which showed positive results.

These findings, taken together with the temporal relationship between the putative drug (acyclovir) and the onset of thrombocytopenia, along with the exclusion of the other most common known causes of thrombocytopenia, established a definitive diagnosis of acyclovir-induced immune thrombocytopenia.

Initial treatment included telling the patient to stop using acyclovir, and giving him 5 units of donor platelets to elevate his platelet count. The patient was also treated with oral prednisone 60 mg daily for possible immune thrombocytopenic purpura (ITP).

### The subsequent visits

One week later, the patient came back for a follow-up visit. We saw that the lesion on the left margin of tongue had regressed obviously. The lesion was of medium texture, had a softer base than before, was not painful on compression, and still had some bleeding spots on the margin (Fig. 2e). The lesions located on the left side of the tongue tip and the right margin of tongue were smaller, and the color had changed from purple to almost white (Fig. 2d and f). His platelet count was elevated to 39 × 10^9^/L (Fig. 3). The third visit: Another 1 week later, the patient came for a second follow-up visit. The lesions located on the left side of the tongue tip and the right margin of tongue had disappeared (Fig. 2g and i). The lesion on the left margin of tongue was smaller, and the color changed from purple to white, with a soft base and no pain on compression. The patient’s platelet count was elevated to 98 × 10^9^/L.

## Discussion

DITP is a challenging clinical problem that is under-recognized, difficult to diagnose, and associated with severe bleeding complications [11]. The first report of DITP was published as early as 1865 [12]. DITP can be triggered by a wide range of medications. There are more than 200 drugs, including some herbal medicines, that have been reported to be causeative of DITP [13, 14]. The incidence of DITP is not well defined; epidemiologic studies performed in the US and Europe showed that approximately 10 persons per million are affected by DITP [15–18]. Despite the low frequency, DITP is important to recognize because of the large number of drugs can be involved and the large number of patients that can be affected [19].

The etiology of DITP is complex. According to the mechanism responsible for the thrombocytopenia, DITP can be divided into two main categories, which are suppression of platelet production and increasing in clearance of peripheral platelets. The former one is caused mostly by myelosuppressive drugs. The latter one can be further divided into three subtypes: nonimmune DITP, immune DITP, and autoimmune DITP [19]. Most DITPs are thought to be caused by the second mechanism, which is mediated by a drug-dependent antibody, and most drugs are thought to cause thrombocytopenia by a drug-dependent immune mechanism. How drugs induce platelet antibodies and how platelets are destroyed by these antibodies are still poorly understood [14].

Patients who experience an unexpected severe thrombocytopenia and an acute drop in platelet levels should be suspected of having DITP. Clinical key features of DITP are: 1) extensive petechiae or ecchymosis, with markedly low blood platelet levels (frequently <10 × 10^9^/L), approximately 3 to 10 days after starting a putative medication [13, 14, 20, 21], and 2) platelet counts return to normal at approximately 7 days after stopping the putative drug (usually in 1–10 days) [22]. Serious bleeding, including intracranial hemorrhage, can occur [23], presenting a challenging diagnostic and management problem.

The diagnosis of DITP is mainly established by the exclusion of all the recognized causes of thrombocytopenia and the temporal association between the administration of the putative drug and the development of thrombocytopenia [10]. A careful, detailed history is crucial to patient evaluation. The patient should be asked specifically about drug exposure that can cause thrombocytopenia, including herbal medicine, tonics, certain foods, drinks, and health supplements. Aster and George have developed the clinical criteria and levels of evidence for diagnosis of DITP [14, 24].

In our case, the patient was given acyclovir as anti-varicella-zoster virus infection treatment. We first considered a bone marrow suppression and immunologic thrombocytopenia, which was associated with viral infections, including human immunodeficiency virus (HIV), hepatitides virus (including hepatitis B and C viruses), Epstein-Barr virus, and cytomegalovirus [25]. The tests for the a forementioned viruses were negative, and varicella-zoster virus is rarely involved in bone marrow suppression. From the complete blood test results, only platelets decreased markedly, and the white cells and hemoglobin concentration remained normal. Consequently, it was unlikely to be viral-induced thrombocytopenia.

As heparin-induced thrombocytopenia (HIT) is the most common cause of a decrease in platelet count [13], we considered excluding it, although the patient did not provide a clear history of having taken heparin. The result of the PF4/heparin antibody test was positive. PF4/heparin antibodies are sensitive and can be seen in some acute diseases, not specifically in HIT. Thus, we performed the SRA assay, which is considered the gold standard of diagnosis of HIT. The result of the SRA array was negative. We decided that HIT was unlikely to be the cause.

Detection of the drug-dependent antiplatelet antibodies in blood can be helpful in diagnosis [22, 26], although waiting for results of this assay is time consuming, and the test may provide false-negative results. We did the assay and got a positive result. There was no detectable immunoglobulin when the patient’s serum was incubated with normal platelets without identification, and there were detectable levels of immunoglobulin when the patient’s serum was incubated with normal platelets in the presence of acyclovir.

Based on the exclusion of other etiologies of thrombocytopenia, platelet count, and the positive antiplatelet antibodies, and with the temporal relationship between the acyclovir and the onset of thrombocytopenia, we could make the diagnosis of acyclovir-induced immune thrombocytopenia.

For most patients, the appropriate treatment is to stop the putative drug, herbal medicine, or food, administering platelet transfusions or other therapies if bleeding is severe [13, 19, 24]. Based on these principles, initial treatment included stopping the use of acyclovir and giving 5 units of donor platelets to for supportive treatment in our case.

## Conclusion

A 67-year-old man developed acyclovir-induced thrombocytopenia after receiving the drug for 10 days VZV infection. Tongue hematoma was the first sign of DITP.

This case highlights that acyclovir could be a causative drug of DITP and that clinicians should be aware of this potential adverse reaction. A hematoma in the oral cavity can be the first complaint, and patients may present to a dentist first. Dentists should be alert to the possibility of this condition.

## Abbreviations

DITP: Drug-induced immune thrombocytopenia
ELISA: Enzyme-linked immunoassay
HIT: Heparin-induced thrombocytopenia
HSV: Herpes simplex virus
ITP: Immune thrombocytopenic purpura
LDH: Lactate dehydrogenase
PF4: Platelet factor 4
SRA: Serotonin-release assay
VZV: Varicella zoster virus
